# Upregulated insulin secretion in insulin-resistant mice: evidence of increased islet GLP1 receptor levels and GPR119-activated GLP1 secretion

**DOI:** 10.1530/EC-12-0079

**Published:** 2013-03-18

**Authors:** L Ahlkvist, K Brown, B Ahrén

**Affiliations:** 1 Department of Clinical Sciences Biomedical Center C11, Lund University SE 22184, Lund Sweden; 2 GlaxoSmithKline, Research Triangle Park Durham, North CarolinaUSA

**Keywords:** GLP1, GLP1R, GPR119, insulin, insulin resistance

## Abstract

We previously demonstrated that the overall incretin effect and the β-cell responsiveness to glucagon-like peptide-1 (GLP1) are increased in insulin-resistant mice and may contribute to the upregulated β-cell function. Now we examined whether this could, first, be explained by increased islet GLP1 receptor (GLP1R) protein levels and, secondly, be leveraged by G-protein-coupled receptor 119 (GPR119) activation, which stimulates GLP1 secretion. Female C57BL/6J mice, fed a control (CD, 10% fat) or high-fat (HFD, 60% fat) diet for 8 weeks, were anesthetized and orally given a GPR119 receptor agonist (GSK706A; 10 mg/kg) or vehicle, followed after 10 min with gavage with a liquid mixed meal (0.285 kcal). Blood was sampled for determination of glucose, insulin, intact GLP1, and glucagon, and islets were isolated for studies on insulin and glucagon secretion and GLP1R protein levels. In HFD vs CD mice, GPR119 activation augmented the meal-induced increase in the release of both GLP1 (AUC_GLP1_ 81±9.6 vs 37±6.9 pM×min, *P*=0.002) and insulin (AUC_INS_ 253±29 vs 112±19 nM×min, *P*<0.001). GPR119 activation also significantly increased glucagon levels in both groups (*P*<0.01) with, however, no difference between the groups. By contrast, GPR119 activation did not affect islet hormone secretion from isolated islets. Glucose elimination after meal ingestion was significantly increased by GPR119 activation in HFD mice (0.57±0.04 vs 0.43±0.03% per min, *P*=0.014) but not in control mice. Islet GLP1R protein levels was higher in HFD vs CD mice (0.8±0.1 vs 0.5±0.1, *P*=0.035). In conclusion, insulin-resistant mice display increased islet GLP1R protein levels and augmented meal-induced GLP1 and insulin responses to GPR119 activation, which results in increased glucose elimination. We suggest that the increased islet GLP1R protein levels together with the increased GLP1 release may contribute to the upregulated β-cell function in insulin resistance.

## Introduction

In fully compensated insulin resistance, there is a sufficient upregulation of insulin secretion whereas in glucose intolerance and type 2 diabetes this upregulation is inadequate (1). Several mechanisms have been suggested to contribute to the upregulated β-cell function in insulin resistance, such as signals generated from nutrient metabolism, hormones, and cytokines (2). We previously suggested that the incretin hormones may also contribute by demonstrating in model experiments that the incretin effect, i.e., the augmented insulin secretion seen after oral vs i.v. glucose, is increased in insulin-resistant mice (3) and that the β-cell responsiveness to intravenous glucagon-like peptide-1 (GLP1) is augmented (3, 4).

The increased incretin effect in insulin resistance raises the question of whether GLP1 secretion from the intestine is increased and may thus be therapeutically leveraged. One approach to test this hypothesis would be to activate G-protein-coupled receptor 119 (GPR119). This receptor is expressed in gut enteroendocrine cells (5), whereby activation has been shown to result in increased release of GLP1 (6, 7). GPR119 protein is also localized to islet β-cells (6), and consequently, GPR119 activation may regulate glycemia both directly by activating islet β-cell insulin secretion (8) and indirectly through release of intestinal incretin hormones.

In this study, we explored, first, whether the increased β-cell responsiveness to GLP1 in insulin-resistant mice is associated with increased islet GLP1 receptor (GLP1R) protein levels. We then examined the potential to improve islet function and glucose tolerance in glucose-intolerant insulin-resistant mice through GPR119 activation by administering a specific GPR119 receptor agonist. As the effects of GPR119 activation on islet α-cells are still unknown, glucagon release was also investigated in view of the importance of glucagon for glycemic dysregulation and reduction of glucagon in therapy of type 2 diabetes (9). We performed the studies in model experiments in mice using the high-fat-fed insulin-resistant mice, which is a well-established model for glucose intolerance (10), and we used the recently developed technique to stimulate islet hormone secretion by mixed liquid meal gavage in mice (11).

## Materials and methods

### GPR119 receptor agonist

In this study, we used a novel small-molecule GPR119 receptor agonist (GSK2041706A, 2-[((1S)-1-(1-[3-(1-methylethyl)-1,2,4-oxadiazol-5-yl]-4-piperidinyl)ethyl)oxy]-5-[4-(methylsulfonyl)phenyl]pyrazine) (GlaxoSmithKline) (12). GSK706A is >100-fold selective for GPR119 receptor over a variety of other receptors, ion channels, and enzymes and possesses an EC_50_=4 nM against the human GPR119 receptor. GSK2041706 (GSK706) is a potent and selective agonists at the rodent and human GPR119 receptor that have been discovered by GlaxoSmithKline (12). For the exploration of the biology associated with activation of GPR119, these synthetic agonists behave similarly. Both agonists are 100-fold more selective for rodent and human GPR119 receptors than a battery of other 7TM receptors, ion channels, and enzymes. In the acute *in vivo* studies, the compound was formulated in a vehicle consisting of 0.5% methocel K15M premium EP hydroxypropyl methylcellulose (HPMC; Dow Chemical, Midland, MI, USA) and 0.1% Tween 80 (Fluka/Sigma–Aldrich) in water. For the *in vitro* experiments, the compound was dissolved in 1% DMSO (Sigma–Aldrich).

### Animals

Female C57BL/6J mice (average 22 g) were obtained from Taconic (Skensved, Denmark). After 1 week of acclimatization, the mice were divided into two groups and fed either a control diet (CD; 10% fat by energy; D12450B Research Diets, New Brunswick, NJ, USA) or a high-fat diet (HFD; 60% fat by energy; D12492, Research Diets) for 8 weeks. Body weight and food intake were monitored once a week. Food and water was provided *ad libitum*. The animals were housed in groups of eight per cage in a temperature-controlled (22 °C) room with artificial lighting maintained on a 12 h light:12 h darkness cycle. All experimental procedures were performed in agreement with the Animal Ethics Committee in Lund, Sweden.

### GPR119 activation *in vivo*

Fasted (5 h) mice were anesthetized with an i.p. injection of midazolam (0.4 mg/mouse, Dormicum, Hoffman-La Roche, Basel, Switzerland) and a combination of fluanisone (0.9 mg/mouse) and fentanyl (0.02 mg/mouse, Hypnorm, Janssen, Beerse, Belgium). The mice were orally gavaged (0.25 ml) with vehicle or GSK706A (10 mg/kg) 10 min before a new gavage with the previously described mixed meal (11). The dose of GSK706A was selected from initial experiments using both lower and higher doses (up to 30 mg/kg) with 10 mg/kg eliciting a maximal response in insulin and glucose. The mixed meal consisted of a mixture of glucose (60% kcal, Sigma), whey protein (20% kcal, SELF Omninutrition, Stockholm, Sweden), and peanut oil (20% kcal, Zeta, Stockholm, Sweden), with total caloric content of 0.285 kcal. Blood samples were collected into heparinized tubes from the retrobulbar, intraorbital, and capillary plexus before and 15, 30, and 60 min after oral gavage for plasma glucose and insulin determination. For glucagon and intact GLP1 measurements, blood samples were collected before and 5, 10, and 20 min after oral challenge into tubes containing for glucagon measurements the protease inhibitor aprotinin (Trasylol; 500 KIE/ml Bayer) or for GLP1 measurements a combination of aprotinin and the dipeptidyl peptidase-4 inhibitor valine pyrrolidide (0.03 mM, Novartis). After collection, all blood samples were immediately centrifuged (4 °C) and plasma was stored (−20 °C) for subsequent analysis.

### GPR119 activation *in vitro*

Mouse islets were isolated from the pancreata of normal and HFD-fed mice by collagenase digestion and handpicked under the microscope. Batches of freshly isolated islets were preincubated in HEPES balanced salt solution containing 125 mmol/l NaCl, 5.9 mmol/l KCl, 1.28 mmol/l CaCl_2_, 1.2 mmol/l MgCl_2_, 25 mmol/l HEPES (pH 7.4), 5.6 mmol/l glucose, and 0.1% fatty acid free BSA (Boehringer Mannheim) at 37 °C during 60 min. Thereafter, islets in groups of three were incubated in 200 μl of the above-described buffer with varying concentrations of glucose. In the first experiment, the direct effect of GSK706A on insulin secretion was determined by incubating isolated islets from mice fed the CD and HFD in different concentrations of glucose (2.8, 5.6, 8.3, 11.1, and 16.7 mmol/l), with or without addition of GSK706A (1 nmol/l). In a second experiment, the effect of non-glucose stimuli on GPR119 activation was determined by incubating isolated islets from mice fed the CD and HFD in different concentrations of glucose (2.8, 11.1 mmol/l) with or without arginine (10 mmol/l) and GSK706A (1 nmol/l). The islets were incubated for 60 min at 37 °C, where after aliquots of 25 or 50 μl buffer in duplicate were collected and stored at −20 °C until analysis of insulin and glucagon respectively.

### Islet GLP1R protein levels

The protein levels of the GLP1R in isolated islets of CD and HFD-fed mice were analyzed by western blot. The islets were homogenized in a buffer containing 150 mmol/l NaCl, 2 mmol/l EDTA, 20 mmol/l Tris–HCl, pH 7.5, 1% Triton X-100, and 0.2% protease inhibitor cocktail (Sigma–Aldrich). The total amount of proteins in each sample was measured using a BCA Protein assay reagent kit (Pierce, Rockford, IL, USA). Aliquots of tissue homogenates containing equal amounts of protein (30 μg) were separated on SDS–PAGE and blotted onto nitrocellulose membranes (Bio-Rad). The membranes were probed with primary antibodies against the GLP1R (53 kDa) (Abcam) and actin (42 kDa) (Abcam). The secondary antibody was a HRP-conjugated goat anti-rabbit IgG antibody (Amersham Pharmacia Biotech). The blots were developed by enhanced chemiluminescence (SuperSignal West Pico, Pierce) and the proteins were detected and quantified using a ChemiDoc XRS+ system with image lab software (Bio-Rad).

### Plasma analysis

Plasma glucose was measured with the glucose oxidase method using 2,2′-azino-bis(3-ethyl-benzothialozine-6-sulphonate) as a substrate with the absorbance measured at 420 nm on a microtiter plate reader (Fluostar/Polarstar Galaxy; BMG Labtechnologies, Offenburg, Germany). Insulin was analyzed with sandwich immunoassay technique (ELISA) using double MABs against insulin (Mercodia, Uppsala, Sweden). Glucagon was measured by RIA (Millipore, Billerica, MA, USA). Levels of intact GLP1 were determined by sandwich immunoassay technique (ELISA) using MABs specific for the active form of GLP1, whereby electrochemiluminescent labeling enables detection of voltage-mediated chemiluminescence in the SECTOR Imager plate reader (Meso Scale Discovery, Gaithersburg, MD, USA).

### Calculations and statistical analysis

Data are reported as mean values ±s.e.m. Statistical significances were assessed by using Student's *t*-test. Suprabasal areas under the curve (AUC) were calculated by the trapezoidal rule for glucose and insulin data during time interval 0–60 min and for glucagon and intact GLP1 during 0–20 min. The glucose elimination constant (*K*_G_) was determined as the glucose elimination rate (% per min) between 15 and 60 min after oral challenge. β-Cell function (0–60 min) was determined as the ratio between AUC insulin and glucose.

## Results

### Body weight and glucose tolerance in CD and HFD-fed mice

Compared to the CD, feeding C57BL/6J mice with a HFD for 8 weeks resulted in an insulin-resistant phenotype, characterized with higher weight gain (11.1±0.5 vs 2.0±0.1 g, *P*<0.001) and elevated fasting glucose (*P*=0.0014) and insulin (*P*<0.001) levels in plasma (Table 1). Furthermore, we observed impaired glucose tolerance (IGT) after mixed meal challenge in the HFD- compared to CD-fed mice, as indicated by higher AUC glucose over 60 min (*P*=0.007) and reduced K_G_ (*P*=0.009) (Table 1). Basal plasma glucagon levels did not differ between control and insulin-resistant mice (Table 1).

### Acute effect of GPR119 activation in CD and HFD-fed mice

**Figure 1:**
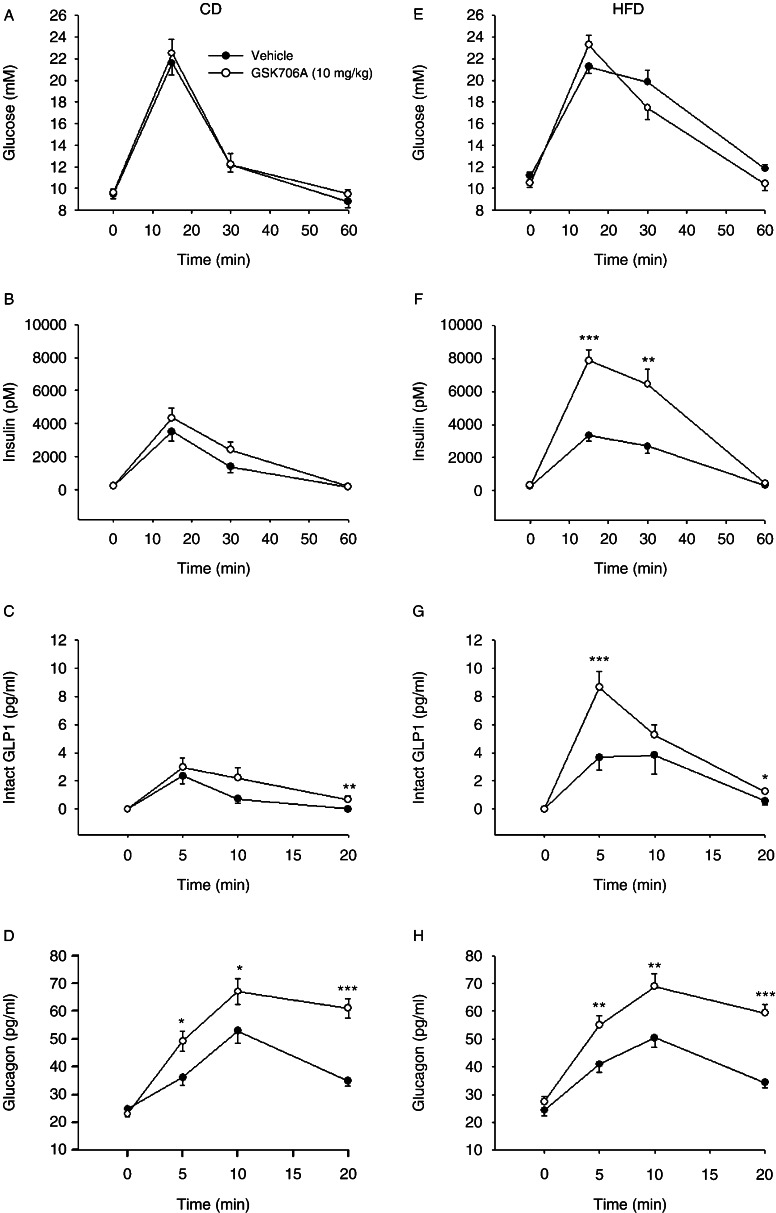
Plasma glucose (A and E), insulin (B and F), intact GLP1 (C and G), and glucagon (D and H) levels after vehicle (closed circle) vs GSK706A (10 mg/kg, open circle) in control diet (CD)- and high-fat diet (HFD)-fed C57BL/6J mice following oral meal challenge. Means±s.e.m. are shown, *n*=9–13 mice per group. *P<0.05, **P<0.01, ***P<0.001.

To determine changes in intestinal GLP1 secretion in insulin resistance, a GPR119 agonist (GSK706A, 10 mg/kg) or vehicle was given orally before (−10 min) an oral liquid test meal to CD and HFD-fed mice. In CD-fed mice, no significant difference in plasma glucose or insulin levels after meal ingestion was observed between gavage of vehicle vs GPR119 agonist (Fig. 1A and B). Nevertheless, β-cell function (insulinogenic index) was higher after GPR119 activation than after vehicle (*P*=0.037) (Table 1). Furthermore, plasma intact GLP1 levels increased after meal ingestion and were significantly augmented by GPR119 after 20 min (0.7±0.2 vs 0.0±0.0 pg/ml, *P*=0.0016) (Fig. 1C). In insulin-resistant mice, the activation of GPR119 markedly increased insulin secretion compared with vehicle (15 min: 7.8±0.6 vs 3.3±0.3 nmol/l, *P*=0.00002; 30 min: 6.4±0.9 vs 2.7±0.4 nmol/l, *P*=0.003) (Fig. 1F), also seen with 60-min AUC data (*P*=0.0004) (Table 1). The insulin secretion due to GPR119 activation in insulin resistance was accompanied by significantly increased plasma levels of intact GLP1 already after 5 min (8.6±1.2 vs 3.7±0.9 pg/ml, *P*=0.004) and levels stayed significantly elevated after 20 min (1.2±0.2 vs 0.6±0.2 pg/ml, *P*=0.05) (Fig. 1G). This was also evident by significantly increased suprabasal 20-min AUC (*P*=0.0011) (Table 1). We also observed a diet-induced difference in plasma GLP1 levels after mixed meal, with higher 20-min AUC levels in high-fat compared with CD feeding (*P*=0.03) (Table 1). The glucose elimination rate was significantly higher after GPR119 activation compared with vehicle (*P*=0.014) (Table 1), although not sufficient to significantly reduce individual glucose levels or AUC glucose (Fig. 1E). β-Cell function was significantly improved after GPR119 activation in HFD mice compared with vehicle (60-min AUC_INS/GLU_; *P*=0.002) (Table 1). Concerning glucagon levels, GPR119 activation increased glucagon levels (Fig. 1D and H) in both CD (20 min AUC: 317±69 vs 650±59 pg/ml×min, *P*=0.0017) and insulin-resistant mice (329±52 vs 608±50 pg/ml×min, *P*=0.0012), compared with vehicle but with no difference between the groups (Table 1).

### GPR119 activation in CD and HFD-fed mouse islets

**Figure 2:**
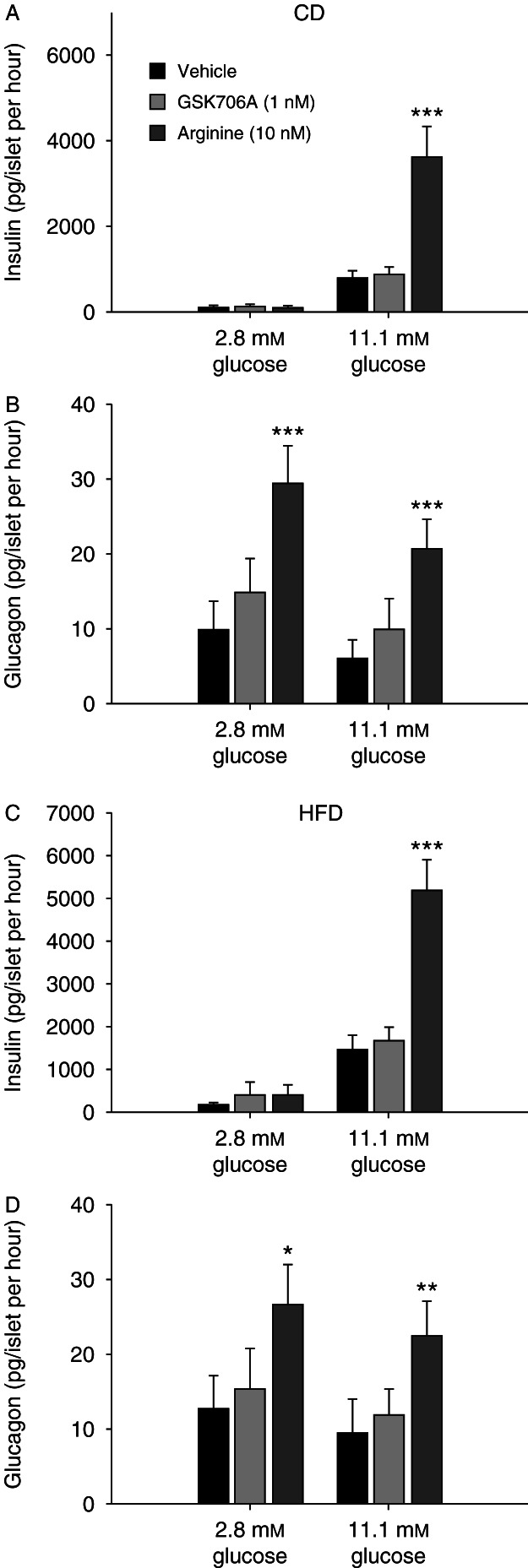
Insulin (A and C) and glucagon (B and D) levels after incubation of isolated islets from control diet (CD) and high-fat diet (HFD) mice in vehicle (black bars), GSK706A (1 nmol/l, light grey bars), or arginine (10 mmol/l, dark grey bars). Means±s.e.m. are shown, *n*=4 mice per group. *P<0.05, **P<0.01, ***P<0.001.

To elucidate whether GPR119 activation had any direct effect on the pancreatic islets, isolated islets from CD and HFD-fed C57BL/6J mice were incubated with the GSK706A compound. First, islets from HFD-fed mice had generally a higher insulin secretion at low (2.8 mmol/l), moderate (8.3 mmol/l), and high glucose (16.7 mmol/l) compared with CD (232±102 vs 97±44 pg/ml per islet, NS), (665±231 vs 275±90 pg/ml per islet, *P*=0.004) and (3355±305 vs 1731±369 pg/ml per islet, *P*<0.001) respectively. By incubating islets from CD and HFD-fed mice with GSK706A (1 nmol/l, 100 nmol/l or 1 μmol/l), we did not observe a significant increase in insulin release at 2.8, 5.6, 8.7, 11.1, or 16.7 mmol/l glucose (data not shown). In a second experiment, the islets were incubated with arginine (10 mmol/l), a potent stimulator of both islet insulin (13) and glucagon (14) secretion, which induced a robust increase in insulin secretion at 11.1 mmol/l glucose (*P*<0.001) in both CD and HFD-fed mouse islets (Fig. 2A and C). Again, no significant effect on insulin secretion was seen after GPR119 activation. Concerning glucagon secretion, GPR119 activation did not result in any significant increase in glucagon, whereas arginine induced a robust glucagon response in islets from both CD and HFD-fed mouse islets compared with vehicle at 2.8 (*P*<0.001 and *P*=0.011 respectively) and 11.1 mmol/l glucose (*P*<0.001 and *P*=0.006 respectively) (Fig. 2B and D).

### GLP1R in CD and HFD-fed mouse islets

**Figure 3:**
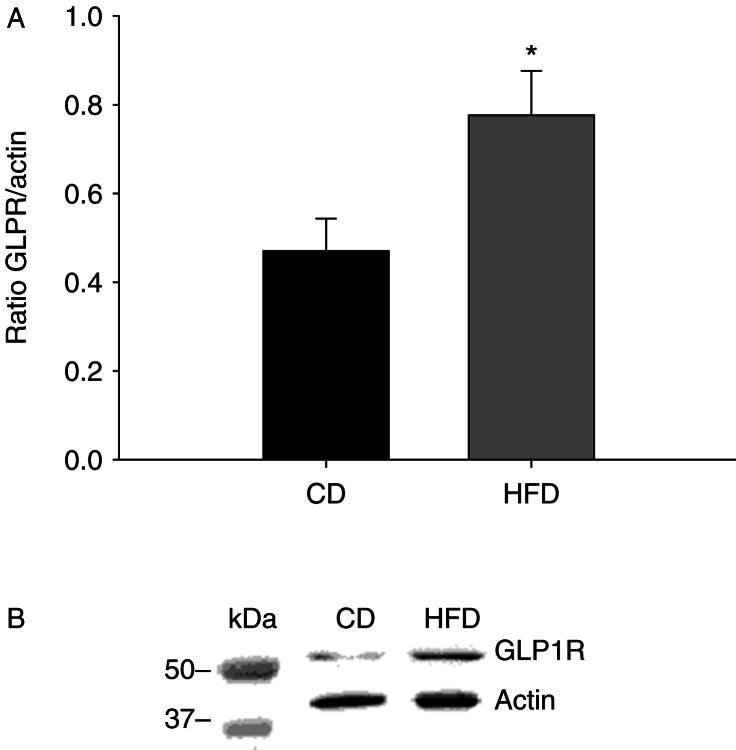
The ratio of GLP1 receptor to actin protein levels (A) and representative western blot (B) in pancreatic islets in control diet (CD, black bar) vs high-fat diet (HFD, grey bar)-fed mice. Means±s.e.m. are shown, *n*=4 mice per group. *P<0.05.

To determine the mechanism behind increased β-cell responsiveness to GLP1 in insulin-resistant mice, diet-induced changes in islet GLP1R protein levels was analyzed with western blot. The ratio of GLP1R to actin protein levels in pancreatic islets was significantly higher in HFD vs CD-fed mice (0.8±0.1 vs 0.5±0.1, *P*<0.05) (Fig. 3).

## Discussion

Insulin resistance leads to a compensatory increase in insulin secretion, which enables sufficient control of blood glucose levels (15). If this adaptation fails, the progressive deterioration of glucose tolerance and type 2 diabetes ensues. In this study, we explored the role of the GLP1 system in the compensatory upregulation of insulin secretion during conditions of insulin resistance in mice and examined the influence of GPR119 activation, which during recent years have been explored as a potential target for the treatment of type 2 diabetes (16). We used the well-characterized HFD-fed mouse model, which is associated with increased body weight, hyperinsulinemia, and glucose intolerance (10). Our main findings are i) that GLP1R expression is increased in pancreatic islets in insulin-resistant mice and ii) that there is an increased GLP1 secretion from the intestine after both high-fat dieting and GPR119 activation. Thus, we suggest that the GLP1 system is upregulated during insulin resistance, through increased responsiveness of islet β-cells to GLP1 and intestinal L-cells to appropriate stimuli, facilitating the compensatory increase in insulin secretion during insulin resistance. This, in turn, may be leveraged by GPR119 activation. We also, for the first time, demonstrate that GPR119 activation increases glucagon secretion in mice.

The GLP1R has been localized in several peripheral tissues (17), and within islets, the GLP1R has been detected mainly in β-cells (18, 19), although the receptor has also been reported in α- and δ-cells (20). The importance of the GLP1R for glucose tolerance has been demonstrated in knockout mice (21), which display an abnormal glucose response to oral glucose challenge in association with reduced insulin secretion. This is the base for the success of incretin therapy in type 2 diabetes (22). In insulin-resistant mice, we have previously demonstrated that the β-cell response to intravenous GLP1 is increased compared with control animals as a sign of increased islet responsiveness to the incretin hormone (3, 4). We here provide mechanistic bases for this effect showing that the increased target cell responsiveness is associated with increased GLP1R protein levels. In line with current data, increased levels of GLP1R mRNA and protein have also recently been found in visceral fat of insulin-resistant individuals (23). Hence, the ability to increase GLP1R protein levels in pancreatic islets during early insulin resistance may be important to facilitate the compensatory increase in β-cell insulin secretion, maintaining normal glucose tolerance.

Previous studies on humans report that individuals with an IGT were found to have an impaired insulinotropic effect of GLP1 (24). Also in type 2 diabetics, incretin action has been shown to be impaired in a manner caused primarily by defects in β-cell responsiveness (25). This may suggest that increased islet GLP1R protein levels, if existent also in humans with IGT or type 2 diabetes, may not be sufficient to counteract the β-cell dysfunction in these subjects. Our finding of upregulated GLP1 response in insulin-resistant mice therefore suggests that the metabolic perturbations due to IGT or diabetes compromise the GLP1 effect resulting in reduced responsiveness in these conditions.

Studies on incretin hormone secretion in individuals with various degrees of glucose tolerance have reported conflicting results. Secretion of GLP1 was found to be impaired in obese subjects with both NGT (26) and IGT (27), whereas another study reported normal GLP1 secretion in obese IGT subjects (28). Although defects in GLP1 secretion have been reported in some type 2 diabetics (29), a recent meta-analysis by Nauck *et al*. (30) concludes that deteriorations in glucose homeostasis can develop in the absence of any impairment in incretin hormone levels. In the current study, we also tested the acute GLP1 response during high fat feeding using the synthetic GPR119 agonist GSK706A. As GPR119 protein has been found in both intestinal and islets endocrine cell, this receptor is an attractive target in diabetes therapy. GPR119 activation has previously been demonstrated to increase GLP1 in wild-type mice with the synthetic GPR119 agonist, AR231453 (31), whereas this effect was lost in *Gpr119* ^−/−^ mice (32). We show that the GLP1 response to both nutrient stimuli and GPR119 activation is enhanced in insulin-resistant mice, leading to elevated levels of GLP1 in plasma. In rats, it was recently shown that a HFD results in elevated GLP1 levels after a mixed meal challenge (33). Also, in another study, GLP1 levels were threefold higher than control in the high-fat-fed canine model of obesity and insulin resistance (34). Thus, these previous reports support the current finding that L-cell GLP1 release may be more responsive to nutrient stimuli when animals are fed a HFD.

The increased GLP1 response to GPR119 activation in association with the increased β-cell levels of the GLP1R in the high-fat-fed mice explains the robust increase in insulin secretion. In pancreatic islets, GPR119 protein has been localized mainly in β-cells (6) and activation of this receptor has been shown to stimulate islet insulin secretion through cAMP-mediated pathways (6, 8). This would suggest that the increased insulin response to GPR119 activation in insulin-resistant mice may also be explained by a more profound direct islet effect of the agonist. However, the current *in vitro* experiments do not support such a notion. On the other hand, as these are static islet incubations, smaller effects of GSK706A such as variations in first-phase insulin release may be masked by the collective insulin secretion during the full incubation time. Thus, in the current study, GPR119 activation had no significant effect on islet insulin release even at very high doses. Therefore, although minor effects on insulin secretion may have been difficult to find in these studies in incubated islets, the marked insulin response to GPR119 activation in insulin-resistant mice is most likely mediated by GLP1.

The elevated intact GLP1 and insulin levels after GPR119 activation in the insulin-resistant mice resulted in increased glucose elimination rate. However, the effect was small and the mechanisms behind the stimulated glucose elimination is not known, as it may be mediated not only by increased insulin levels but also by direct effects on hepatic glucose production and glucose kinetics, which were not studied in detail in the current study. Conversely, plasma glucose levels were not reduced by GSK706A at any of the individual time points and, similarly, the AUC for glucose was not lowered. Thus, no change in glucose tolerance but increased insulin levels occurred after GPR119 activation, which may suggest that insulin may be less effective, which also needs to be studied in more detail. Therefore, our overall and main conclusion based on these results is that GPR119 activation seems a weak tool to improve glycemia in the setting of insulin resistance.

The failure of GPR119 activation to lower glucose below baseline may be explained by the concomitant increase in plasma glucagon levels after GPR119 activation. The mechanism behind the glucagonotropic action of GPR119 activation remains to be established. As GLP1 and insulin are potent inhibitors of glucagon secretion (35, 36), it is possible that the GSK706A component directly affects islet α-cells. In rodent islets, GPR119 protein has been detected in α-cells (6, 37), however in relatively low concentration compared to β-cells. Concordantly, the current *in vitro* experiments do not indicate that GPR119 activation has any direct effect on islet glucagon release, and thus, not likely the main contributor to the increased *in vivo* plasma glucagon levels. As GPR119 protein levels is found not only in the gut L-cells, which produce GLP1, but also in other enteroendocrine cells, it is possible that receptor activation indirectly affects islet glucagon release via other gut hormones than GLP1. For example, GPR119 has been found in K-cells (38), and receptor activation has been shown to increase glucose-dependent insulinotrophic peptide (GIP) levels (6, 31, 32). GIP has known glucagonotropic effects, however, previously only demonstrated during fasting and euglycemic conditions in healthy humans (39, 40), and at glucose concentrations below 5.5 mM in the perfused rat pancreas (41). On the other hand, studies on type 2 diabetics have pointed out that there is a lack of glucagon suppression after an oral glucose load, however preserved suppression after an isoglycemic i.v. glucose infusion (IIGI) (42, 43), pointing to gut factors as potential mediators of dysregulated postprandial glucagon secretion in diabetes. In fact, in type 2 diabetics, Lund *et al* have showed that GIP infusion during the IIGI results in hypersecretion of glucagon (44). Hence, in the setting of insulin resistance, it can therefore not be concluded whether a GPR119-mediated increase in GIP levels is causing the rise in glucagon after the current meal challenge. As excessive α-cell glucagon secretion is an important contributor to hyperglycemia during type 2 diabetes, the ability to lower glucagon secretion is one of the main therapeutic advantages of incretin-based therapy (45). Thus, the discrepancy between GLP1 and glucagon plasma levels in this study suggests that GPR119-based therapy is likely most effective as part of combination therapy. Indeed, paired with a DPP-4 inhibitor, which blocks the enzymatic inactivation of GLP1 (46), there is evidence to support that the glucose-lowering potential of GSK706A could be enhanced (47). Furthermore, as antagonism of the glucagon receptor has been shown to improve islet function in mice with insulin resistance induced by a HFD (48), GSK706A could suggestively be combined with an inhibitor of glucagon action to achieve more potent glucose-lowering effects.

In conclusion, we suggest that the normal adaptive response of the β-cell to insulin resistance involves an upregulation of the incretin system manifested as both increased islet GLP1R protein levels and enhanced GPR119-activated GLP1 secretion, resulting in improved β-cell insulin secretion. However, no major changes in glucose tolerance are observed. We also show that GPR119 activation stimulates glucagon secretion, which increases glucagon levels. Therefore, a further development of an anti-diabetic effect of GPR119 activation may require combination with glucagon antagonism. These results thus provide both a mechanistic basis for increased incretin effect and β-cell sensitivity to GLP1 in insulin-resistant mice and a rationale for improving islet function by GPR119 activation.

## Author contribution statement

L Ahlkvist contributed with the design of the study, undertaking of the experiments, analyses and statistics, interpretation and discussion of the data, and writing the manuscript. K Brown contributed with the design of the study and gave comments to the manuscript. B Ahrén contributed with design of the study, interpretation and discussion of the data, and writing the manuscript.

## Figures and Tables

**Table 1:** Weight gain, fasting and dynamic intact GLP1, islet hormones and β-cell function after vehicle (A) vs GSK706A (B) in control diet (1) and high-fat diet (2) fed C57BL/6J mice following oral meal challenge.

	**CD vehicle** ^(1A)^	**CD GSK706A** ^(1B)^	**HFD vehicle** ^(2A)^	**HFD GSK706A** ^(2B)^
Weight gain (8 weeks, g)	2.0±0.1		11.1±0.5 *P*<0.001	
Fasting glucose (mmol/l)	9.5±0.3		11.0±0.2 *P*=0.0014	
Fasting insulin (pmol/l)	179±11		322±23 *P*<0.001	
Fasting glucagon (pg/ml)	24.0±1.0		25.9±1.3 NS	
K_G_ (% per min)	0.65±0.07	0.61±0.06 NS	0.43±0.03 (^†^ 1A/2A)	0.57±0.04 *P*=0.014
AUC_GLUCOSE__×__60 min_ (mmol/l)	233±22	251±35 NS	355±33 (^†^ 1A/2A)	347±25 NS
AUC_INSULIN__×__60 min_ (nmol/l)	76±16	112±19 NS	101±13	253±29 *P*<0.001
AUC_GLP1__×__20 min_ (pg/ml)	20.1±3.8	36.7±7.9 NS	34.6±4.8 (* 1A/2A)	80.6±9.6 *P*=0.0014
AUC_GLUCAGON__×__20 min_ (pg/ml)	317±69	650±59 *P*=0.0017	329±52	608±50 *P*=0.0017
AUC_INS_/AUC_GLU__×__60 min_	315±60	513±67 *P*=0.037	304±40	760±108 *P*=0.002

Means±s.e.m. are shown, *n*=9–13 mice per group. K_G_ is defined as the glucose elimination rate 15–60 min during oral challenge. β-Cell function is represented by the ratio AUC_INSULIN_ to AUC_GLUCOSE_. AUC, suprabasal area under the curve. *P<0.05, ^†^ *P*<0.01 for differences between vehicle in the two diet groups.
